# The miRNAome of ramie (*Boehmeria nivea* L.): identification, expression, and potential roles of novel microRNAs in regulation of cadmium stress response

**DOI:** 10.1186/s12870-018-1561-5

**Published:** 2018-12-22

**Authors:** Kunmei Chen, Yongting Yu, Kai Sun, Heping Xiong, Chunming Yu, Ping Chen, Jikang Chen, Gang Gao, Aiguo Zhu

**Affiliations:** Institute of Bast Fiber Crops, Chinese Academy of Agricultural Sciences, Changsha, 410205 China

**Keywords:** Ramie (*Boehmeria nivea* L.), miRNA, Cadmium stress, Target gene, Q-PCR

## Abstract

**Background:**

MicroRNAs (miRNAs) regulate numerous crucial abiotic stress processes in plants. However, information is limited on their involvement in cadmium (Cd) stress response and tolerance mechanisms in plants, including ramie (*Boehmeria nivea* L.) that produces a number of economic valuable as an important natural fibre crop and an ideal crop for Cd pollution remediation.

**Results:**

Four small RNA libraries of Cd-stressed and non-stressed leaves and roots of ramie were constructed. Using small RNA-sequencing, 73 novel miRNAs were identified. Genome-wide expression analysis revealed that a set of miRNAs was differentially regulated in response to Cd stress. In silico target prediction identified 426 potential miRNA targets that include several uptake or transport factors for heavy metal ions. The reliability of small RNA sequencing and the relationship between the expression levels of miRNAs and their target genes were confirmed by quantitative PCR (q-PCR). We showed that the expression patterns of miRNAs obtained by q-PCR were consistent with those obtained from small RNA sequencing. Moreover, we demonstrated that the expression of six randomly selected target genes was inversely related to that of their corresponding miRNAs, indicating that the miRNAs regulate Cd stress response in ramie.

**Conclusions:**

This study enriches the number of Cd-responsive miRNAs and lays a foundation for the elucidation of the miRNA-mediated regulatory mechanism in ramie during Cd stress.

**Electronic supplementary material:**

The online version of this article (10.1186/s12870-018-1561-5) contains supplementary material, which is available to authorized users.

## Background

Cadmium (Cd) is one of the most hazardous pollutants, which causes severe toxicity in plants [1, 2]. Because of its high solubility in water, it is promptly taken up by plants, which constitutes the main entry pathway into the food chain, and can cause serious problems to human health [3]. Even at low concentrations, the uptake of Cd by plant roots and its transport to the vegetative and reproductive organs have a negative effect on mineral nutrition and homeostasis during the growth and development of plant shoots and roots [4, 5]. The elevated levels of Cd in agricultural soils, as a consequence of mining activities, industrial emissions, and the application of sewage sludge or phosphorous fertiliser, is a significant environmental problem and is considered a major threat to humans [6]. In China, for example, approximately 2.8 × 10^5^ ha of farmland is polluted by Cd [7].

Ramie (*Boehmeria nivea* L.) is a perennial herb with high shoot biomass (harvested more than three times per year) and a strong root system [8]. As one of the most important natural fibre crops, ramie has been widely cultivated in southern China for more than 5000 years. Owing to its excellent characteristics, including smooth texture, long fibre length, and excellent tensile strength, ramie fibre is widely used in China, Southeast Asia, and Pacific rim countries. Ramie can effectively accumulate and strongly tolerate certain heavy metals, such as Cd [9, 10], lead [11], and arsenic [8], from contaminated soil. Ramie is capable of absorbing approximately 0.19 to 1.09 kg Cd (ha^− 1^ y^− 1^) from Cd-contaminated farmlands [7]. Because ramie fibres are mainly used as textile materials, the potential hazard of introducing toxic metals into the food chain is reduced. Therefore, ramie is an ideal crop for the phytoremediation of mildly or moderately Cd-polluted areas.

Understanding the expression and regulation of Cd-responsive genes is the first step in dissecting the genetic and molecular basis of Cd high accumulation in plants. However, little research has been performed on deciphering the molecular mechanism of Cd tolerance in ramie. Regulation of gene expression can be achieved at transcriptional, post-transcriptional, or translational levels. Recently, the post-transcriptional regulation of genes by a group of small RNAs (sRNAs), including microRNAs (miRNAs) and small interfering RNAs (siRNAs), revealed a new mechanism for plant development and their tolerance to environmental stresses [12]. The miRNAs processed from single-stranded RNA precursors that are capable of forming imperfectly complementary hairpin structures through the RNase III enzyme DICER-LIKE1 (DCL1) or DCL4, are known to base-pair their target messenger RNAs (mRNAs) to repress their translation or to induce their degradation in organisms [13]. A set of miRNAs is involved in determining the responses of plants to a wide range of stresses, including heavy metals [14], drought [15], salinity [16], and heat [17] stresses. For example, several miRNAs responding to mercury were found in *Medicago truncatula* [14], and several putative small RNAs responding to Cd were recently identified in rice [18]. A total of 13 conserved miRNAs, representing nine families were isolated from *Brassica napus* L. and showed different responses to Cd [19]. These results show that miRNAs are involved in plant responses to Cd. However, little is known about the Cd-responsive miRNAs in ramie.

High-throughput sequencing technology is a powerful tool that allows concomitant sequencing of millions of genomic signatures from a single tissue [14, 20]. Small RNAs obtained from HiSeq™ (Illumina, San Diego, CA, USA) deep sequencing cover nearly every kind of RNA, including miRNA, siRNA, Piwi-interacting RNA, ribosomal RNA (rRNA), transfer RNA (tRNA), small nuclear RNA (snRNA), and small nucleolar RNA (snoRNA). Thus, many novel miRNAs involved in plant stress responses can be revealed via this technology. We, therefore, used this technology in the present study to recover sRNAs from leaves and roots of ramie either not exposed to Cd or exposed to 10 mg L^− 1^ Cd with the aim of identifying miRNAs involved in Cd stress response. The expression of the identified miRNAs was investigated in the unique ramie variety, “Zhongzhu NO.1”, under normal and Cd stress conditions. The target genes of the miRNAs related to the Cd stress response in ramie were predicted. The identifying novel candidate miRNAs that contribute to Cd tolerance would allow plant breeders to further improve Cd tolerance and increase Cd absorption in ramie.

## Results

### Phenotypic response to cd-treatment

**Fig. 1:**
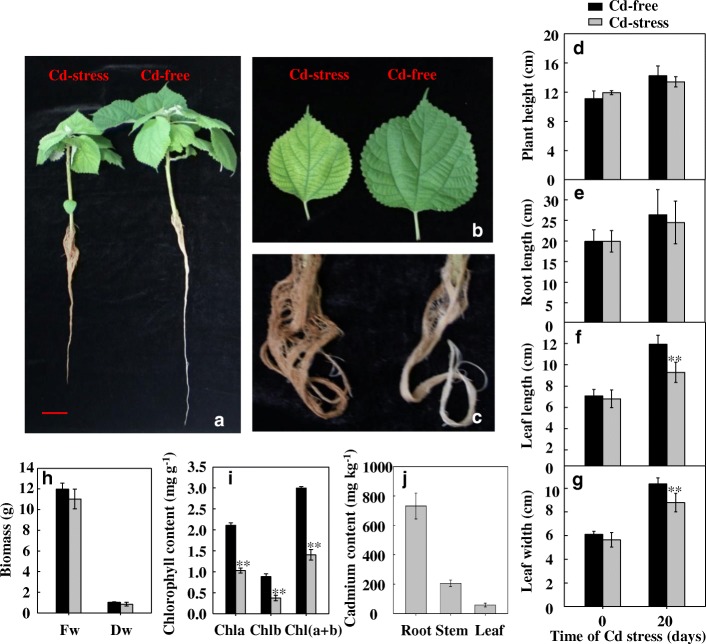
Phenotypic changes in ramie plants under cadmium (Cd) stress (treated with 10 mg L^− 1^ CdCl_2_). **a**–**c** Phenotypic changes in the whole plant, leaf, and root, respectively. The red bar indicates a scale of 2 cm. **d**–**h** Changes in plant height, root length, leaf length, leaf width, and biomass. Fw, fresh weight of the whole plant. Dw, dry weight of the whole plant. **i** Chlorophyll content in the fourth leaf from the top of ramie plants under Cd stress for 20 days. Chla, chlorophyll *a*; Chlb, chlorophyll *b*; Chl(a + b), chlorophyll *a* and *b*. **j** Cd content in the root, stem, and leaf of ramie plants under Cd stress for 20 days. For **d**-**g**, data are means ± standard deviation (SD) of three replicates. Each replicate included 10 individual plants. ** denotes significantly different from the Cd-free (control) at *P* < 0.01

As shown in Fig. 1, the growth of plants exposed to Cd stress was inhibited compared to that of control plants. The length and width of leaves in plants exposed to Cd stress, as well as their biomass, were smaller than that of control plants. In addition, in plants exposed to Cd stress, roots became brown, and leaves showed chlorosis and, accordingly, had lower chlorophyll content than in control plants. The Cd absorbed by ramie was mainly distributed in roots, followed by stems and leaves.

### Sequence analysis and mapping

To identify the miRNAs responsive to Cd stress in ramie, the four sRNA libraries constructed from CL, TL, CR, and TR samples were sequenced on the Illumina HiSeq 2500 platform. The CL, TL, CR, and TR libraries produced 32,557,284, 34,638,385, 29,537,747, and 21,208,181 raw reads, respectively. After filtering out contaminant and low-quality data, the CL, TL, CR, and TR libraries contained 12,342,160, 14,024,186, 19,725,262, and 14,987,053 clean reads, respectively (Additional file 1: Table S1).

To sort sRNAs into categories, all clean reads from the four libraries were aligned against the Silva, GtRNAdb, Rfam, and Repbase by Bowtie, and classified into five categories (rRNA, snRNA, snoRNA, tRNA, and miRNA) based on their matches to these databases (Table 1). Unannotated RNAs, including miRNAs, were mapped onto a previous transcriptome assembly of ramie [22] using miRDeep2: 1,941,679 (26.21%) reads from CL, 1,787,093 (23.03%) reads from TL, 657,206 (9.48%) reads from CR, and 550,022 (11.62%) reads from TR (Additional file 1: Table S1).

**Table 1:** Summary of sRNA sequencing and classification of sequences in the CL, TL, CR and TR libraries

	CL		TL		CR		TR	
	reads	Percentage (%)	reads	Percentage (%)	reads	Percentage (%)	reads	Percentage (%)
rRNA	4,507,417	36.52	rRNA	5,855,373	11,719,819	59.42	9,433,186	62.94
snRNA	2681	0.02	4941	0.04	7509	0.04	7048	0.05
snoRNA	61	0.00	143	0.00	620	0.00	427	0.00
tRNA	417,223	3.38	390,019	2.78	1,052,324	5.33	802,179	5.35
Repbase	6242	0.05	14,359	0.10	9231	0.05	8967	0.06
Unannotated	7,408,536	60.03	7,759,351	55.33	6,935,759	35.16	4,735,246	31.60
Total	12,342,160	100.00	14,024,186	100.00	19,725,262	100.00	14,987,053	100.00

CL, Cd-free leaf (control); TL, Cd-treated leaf; CR, Cd-free root (control); TR, Cd-treated root. rRNA, ribosomal RNA; snRNA, intranuclear small RNA; snoRNA, nucleolar small RNA; tRNA, transport RNA; Repbase, repetitive sequence

**Fig. 2:**
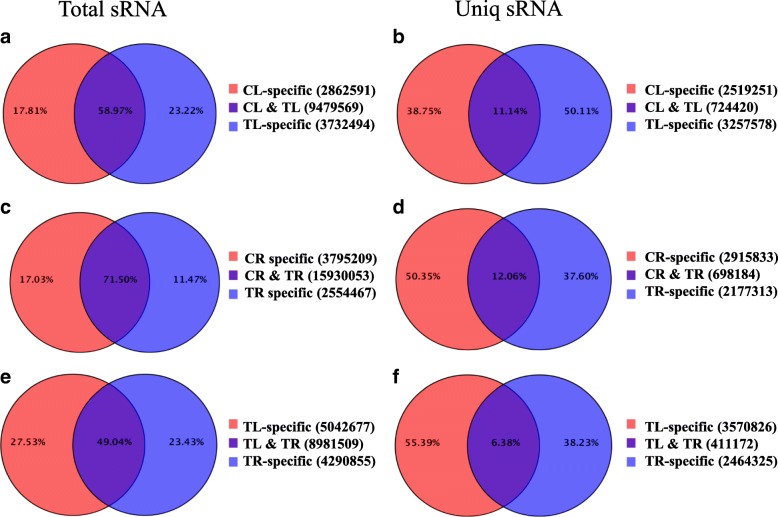
Specific reads within each library and reads common between CL/TL, CR/TR, and TL/TR libraries. **a** Distribution of total clean reads in CL and TL libraries. **b** Distribution of unique reads in CL and TL libraries. **c** Distribution of total clean reads in CR and TR libraries. **d** Distribution of unique reads in CR and TR libraries. **e** Distribution of total clean reads in TL and TR libraries. **f** Distribution of unique reads in TL and TR libraries. CL, Cd-free leaf (control); TL, Cd-treated leaf; CR, Cd-free root (control); TR, Cd-treated root

After filtering out redundant clean reads, the total number of sRNAs in leaf (CL and TL) and root (CR and TR) libraries was 16,074,654 and 22,279,729, respectively, whereas the total number of unique sequences was 6,501,249 and 5,791,330, respectively. The CL and TL libraries and the CR and TR libraries shared 58.97 and 71.50% common reads, respectively, and a low proportion of unique reads (11.14% in leaf libraries and 12.06% in root libraries; Fig. 2). The number of CL-specific and TL-specific unique reads was 2,519,251 (38.75%) and 3,257,578 (50.11%), respectively, whereas the number of CR-specific and TR-specific unique reads was 2,915,833 (50.35%) and 2,177,313 (37.60%), respectively. These results indicate that Cd stress induced the expression of some unique sRNAs in the leaves and repressed that of other sRNAs in the roots.

**Fig. 3:**
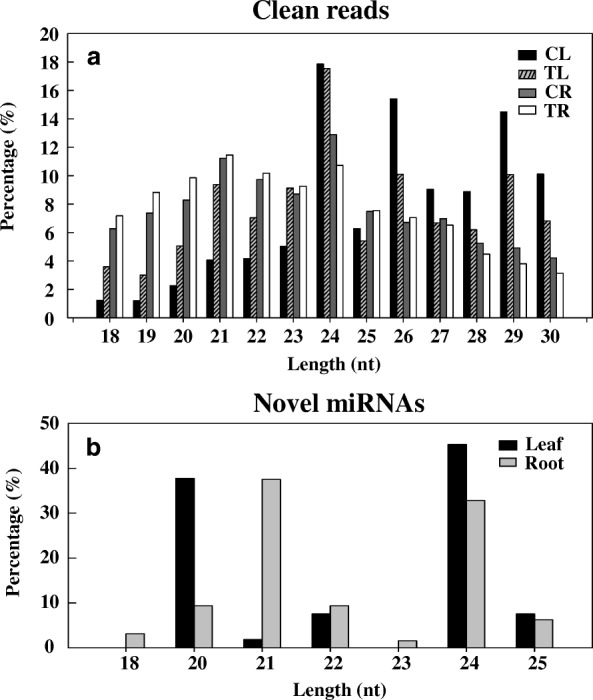
Length distribution of total small RNA (**a**) and novel miRNA (**b**) sequences in different libraries. CL, Cd-free leaf (control); TL, Cd-treated leaf; CR, Cd-free root (control); TR, Cd-treated root

According to the size distribution of the clean reads obtained from each library (Fig. 3a), sRNAs with 24 nt were most frequent in leaves, accounting for 17.86 and 17.54% of the reads in CL and TL libraries, respectively, whereas sRNAs with 21 nt were most frequent in roots, accounting for 11.23 and 11.46% of the reads in CR and TR libraries, respectively. The total number of 24-nt sRNAs in CL was lower than that in TL, but the total number of 21-nt sRNAs in CR was higher than that in TR, implying that more 24 nt sRNA loci in leaves were repressed by Cd stress but more 21 nt sRNA loci in roots were induced.

### Identification of novel miRNAs in response to cd stress

The clean reads mapped onto the ramie transcriptome were used to predict novel miRNAs using miRDeep2. To identify novel Cd stress-responsive miRNAs, we compared clean reads between the CL and TL libraries and between the CR and TR libraries. All the novel miRNA sequences were named in the form of novel-mir-number. A total of 73 novel miRNAs, including 45 conserved and 28 unconserved miRNAs, were identified in the four libraries (Additional file 2: Sheet 1). The total number of novel miRNAs in leaf and root libraries was 53 and 64, respectively. Of the 73 novel miRNAs, 44 were found in leaf and root libraries, whereas 9 and 20 novel miRNAs were unique to the leaf and root libraries, respectively. The novel miRNAs in the leaf and root libraries were mainly 24-nt (32.88% of the 73 novel miRNAs) and 21-nt (32.88%) long, respectively (Fig. 3b). Seven of the 73 novel miRNAs matched to the MIR408_2, MIR535, MIR156, MIR172, MIR408, MIR319, MIR171_1, MIR171_2, and MIR159 families (Additional file 2: Sheet 2).

### Differentially expressed novel miRNAs under cd stress

**Fig. 4:**
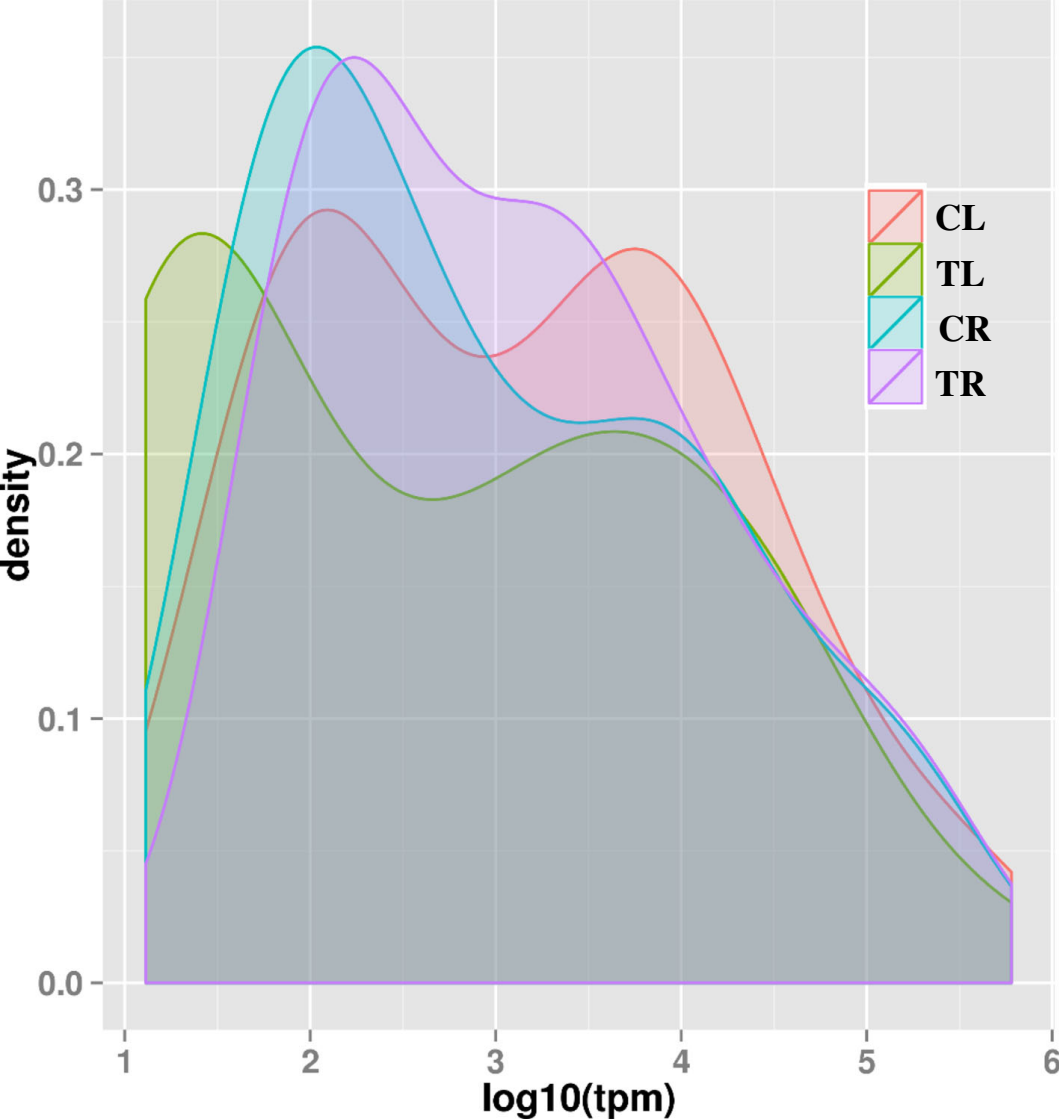
Expression abundance of all novel miRNAs in CL, TL, CR, and TR libraries. TPM, terms of transcripts per million. CL, Cd-free leaf (control); TL, Cd-treated leaf; CR, Cd-free root (control); TR, Cd-treated root

Figure 4 showed the expression abundance of novel miRNAs in Cd stressed and non-stressed leaves and roots of ramie. To determine the differentially expressed patterns of the novel miRNAs in response to Cd stress, we compared the abundance of miRNA reads between the CL and TL libraries and between the CR and TR libraries. Among the 38 novel miRNAs that were differentially expressed under Cd stress, 20 were common to leaf and root, 13 were unique to leaf, and five were unique to root (Additional file 2: Sheet 3). In the leaves, Cd treatment up-regulated 10 novel miRNAs and down-regulated 23, while in the roots, 14 novel miRNAs were up-regulated and 11 were down-regulated.

### Prediction and annotation of genes targeted by the novel miRNAs

Sequences of the 73 novel miRNAs were used as queries against the ramie transcriptome to identify putative target genes of miRNAs. All the novel miRNAs had potential target-gene candidates, and the total number of predicted genes was 426. Functional annotations of these genes were performed by sequence comparison against the NCBI NR, Swiss-Prot, GO, COG, KEGG, KOG, and Pfam databases, and 234 of the target genes were annotated: 59 in the leaves and 175 in the roots (Additional file 2: Sheet 4 and Sheet 5).

For the CL and TL libraries, GO enrichment analysis showed that there were 26 target genes of the differentially expressed miRNAs, which were assigned to 22 GO terms (Table 2), 13 within the biological process category, six within the cellular component category, and three within the molecular function category. Of the 26 target genes, 12 were involved in binding, including magnesium ion binding (1) and zinc ion binding (1), and six were involved in the response of plants to abiotic stress (Additional file 2: Sheet 4). The KEGG analysis identified seven genes as targets of the differentially expressed miRNAs, which were involved in 11 pathways, including arginine and proline metabolism, carbon fixation in photosynthetic organisms, glutathione metabolism, and base excision repair, among others (Additional file 1: Table S2). These pathways are involved in translation, ribosomal structure, and biogenesis (ko03013), amino acid transport and metabolism (ko00590, ko00330, ko00430, ko00460, and ko00480), RNA processing and modification (ko03015), and lipid transport and metabolism (ko00561).

**Table 2:** GO categories of genes targeted by cadmium stress-responsive miRNAs in leaf of ramie

GO_classify1	GO_classify2	DE miRNA Target
cellular component	extracellular region	1
	cell	8
	membrane	4
	organelle	7
	membrane part	1
	cell part	8
molecular function	nucleic acid binding transcription factor activity	1
	catalytic activity	15
	binding	12
biological process	reproduction	1
	metabolic process	17
	cellular process	10
	reproductive process	2
	biological adhesion	1
	signaling	2
	multicellular organismal process	3
	developmental process	3
	single-organism process	13
	response to stimulus	6
	localization	1
	biological regulation	4
	cellular component organization or biogenesis	1

“DE” denotes differential expression

Regarding the CR and TR libraries, 86 and 33 target genes of the differentially expressed miRNAs were found in GO and KEGG analyses, respectively. The 86 target genes were assigned to 30 GO terms that were classified into three categories: biological process with 16 terms, and cellular component and molecular function with seven terms each (Table 3). Of the 86 genes, 13 were involved in metal ion binding, five were involved in transporter activity, and 14 were related to abiotic stress response. The KEGG analysis showed that 21 target genes of the differentially expressed miRNAs were involved in 25 pathways, including arginine and proline metabolism, starch and sucrose metabolism, carotenoid biosynthesis, base excision repair, and tricarboxylic acid cycle, among others (Additional file 1: Table S3).

**Table 3:** GO categories of genes targeted by cadmium stress-responsive miRNAs in root of ramie

GO_classify1	GO_classify2	DE miRNA Target
cellular component	cell	31
	membrane	29
	macromolecular complex	1
	organelle	27
	organelle part	12
	membrane part	13
	cell part	31
molecular function	catalytic activity	48
	receptor activity	2
	transporter activity	5
	binding	40
	electron carrier activity	1
	enzyme regulator activity	2
	molecular transducer activity	2
biological process	reproduction	6
	immune system process	1
	metabolic process	57
	cellular process	46
	reproductive process	6
	biological adhesion	2
	signaling	4
	multicellular organismal process	7
	developmental process	7
	growth	1
	response to stimulus	18
	localization	13
	establishment of localization	12
	multi-organism process	1
	biological regulation	13
	cellular component organization or biogenesis	3

“DE” denotes differential expression

### Validation of expression of novel miRNAs and predicted target genes by q-PCR

**Fig. 5:**
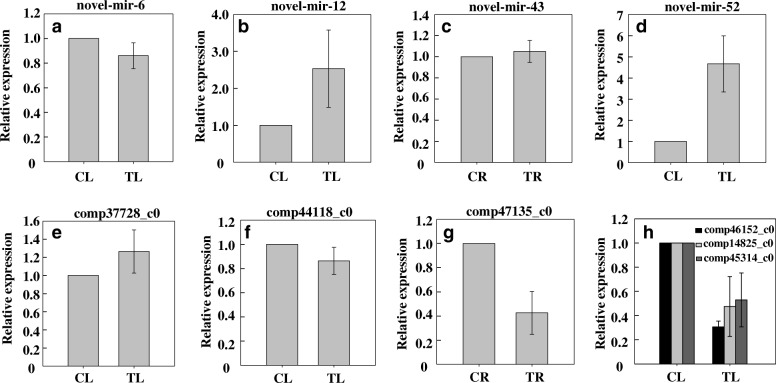
q-PCR analysis of randomly selected novel miRNAs and their target genes in ramie. **a**-**d** Expression of novel-mir-6, novel-mir-12, novel-mir-43 and novel-mir-52. **e**-**h** Expression of target genes. comp37728_c0, target gene of novel-mir-6; comp44118_c0, target gene of novel-mir-12; comp47135_c0, target gene of novel-mir-43; comp46152_c0, comp14825_c0, comp45314_c0, target genes of novel-mir-52. CL, Cd-free leaf (control); TL, Cd-treated leaf; CR, Cd-free root (control); TR, Cd-treated root. For Cd stress experiment, plants were treated with 10 mg L^− 1^ CdCl_2_ for 20 days. The 2^-ΔΔCt^ method was used to calculate the relative expression. The expression in CL and CR was used as the control, and their value was set to 1. Data are means ± standard deviation (SD) of three replicates

Four novel miRNAs (novel-mir-6, novel-mir-12, novel-mir-52, and novel-mir-43) were randomly selected for q-PCR to test the reliability of sRNA sequencing results. The expression patterns of the selected miRNAs in leaves and roots of plants exposed to Cd treatment were similar in the deep sequencing and q-PCR analysis (Fig. 5a-d, Additional file 2: Sheet 3). To investigate the relationship between the expression of miRNAs and their target genes, the expression levels of the target genes (comp37728_c0, comp44118_c0, comp46152_c0, and comp47135_c0, comp14825_c0, comp45314_c0) of the four miRNAs were assessed in leaves or roots of ramie by q-PCR. The expression of the selected target genes was inversely related to that of the corresponding miRNAs (Fig. 5).

## Discussion

Ramie is an ideal plant for the remediation of Cd polluted soil because of its high Cd tolerance and absorption ability. Many studies have been conducted to decipher the physiological and biochemical mechanisms underlying Cd tolerance and uptake in ramie. However, the molecular mechanisms responsible for such tolerance are not known. Although miRNAs play important regulatory roles in the response of plants to heavy metal stress [14], the miRNA response to Cd stress had, hitherto, not been determined in ramie. In the present study, novel miRNAs responses to Cd stress were identified in ramie using high-throughput sequencing of the elite ramie variety, Zhongzhu NO.1, and their expression and target genes were discussed.

Under Cd stress, the root library (TR) had a higher number of sRNAs (18,484,520) than that in the leaf library (TL; 13,212,063) (Fig. 2), which might be due to the root being the first tissue to undergo Cd stress, thereby stimulating more response procedures that result in the expression of more sRNAs. However, the number of unique sRNAs was lower in the TR library than in the TL library (2,875,497 vs. 3,981,998; Fig. 2), indicating that some root sRNAs were inhibited and some leaf sRNA were induced under Cd exposure. This implies that Cd stress can regulate the expression of sRNAs, inducing the expression of some and repressing those of others. In fact, Cd stress is one of the most important abiotic stresses and has been shown to change sRNA expression profiles in plants, such as *Triticum aestivum* L. [26] and *Solanum torvum* Sw. [27]. These Cd stress-responsive sRNAs, especially miRNAs, play crucial roles in acclimation to Cd stress and in mediation of signalling responses to Cd stress. In plants, a substantial amount of the total sRNA abundance and sequence diversity is linked to 21- to 24-nucleotide siRNAs [23]. In the present study, most sRNAs in the leaf libraries (CL and TL) were distributed within the 24-nt length class, whereas most sRNAs in the root libraries (CR and TR) were the 21-nt length class (Fig. 3a), indicating a functional differentiation between the leaves and roots of ramie.

As the complete genome sequence of ramie is not yet available, we mapped the sequenced sRNAs to the ramie transcriptome sequence [22]. However, only a small proportion of the sRNAs (9.48 and 11.62% unique sRNAs from the CR and TR libraries, respectively) were mapped to the transcriptome of ramie. In plants, the majority of miRNA genes are located in intergenic regions [28]. For instance, among the 26 miRNA sequences identified in *Ectocarpus* sp., 17 were found to be located in the introns, eight in the intergenic regions, and one was located antisense to a transposable element [29]. In *Gossypium arboretum* L., 19 miRNAs originated from 32 different intronic regions of different annotated protein coding genes, including their isoforms and copies [30]. It is reasonable to hypothesize that a high proportion of miRNA loci is also located in the pro moter or UTR sequences, intergenic regions and/or introns rather than in the exons of ramie, resulting in the small proportion of sRNAs mapped to the ramie transcriptome.

Because miRNAs function by regulating their target genes (inhibiting translation or degrading the target mRNA) [31], identifying the potential target genes of miRNAs is crucial for understanding the miRNA-mediated regulation mechanism during Cd stress. In the present study, 426 genes were potentially targeted by the 73 novel miRNAs. When plants are subjected to biotic or abiotic stress, the expression of a range of genes changes. Based on the results obtained here, the potential target genes of miRNAs respond to cadmium ion (GO:0046686), oxidative stress (GO:0006979), heat (GO:0009408), high light intensity (GO:0009644), hydrogen peroxide (GO:0042542), freezing (GO:0050826), and hyperosmotic salinity (GO:0042538) stresses. In whole plants, the roots are the primary sites for the uptake of heavy metals. Consequently, the content of Cd in the roots of ramie was much higher than that in the leaves (Fig. 1). Gene expression patterns change in response to toxic elements and, in the present study, most of the target genes of miRNAs in the root library were involved in metal ion binding. After sensing the heavy metal, plant cells activate specific genes to counteract the stress stimuli. A signal transduction cascade is therefore responsible for the differential gene regulation.

Multiple sets of metal stress-responsive miRNAs and their target genes have been identified in plants [32–34]. Plant miRNAs usually mediate gene silencing at the post-transcriptional level, which entails the endonucleolytic cleavage and/or translational repression of a target mRNA [35]. The expression of targets (comp37728_c0, comp44118_c0, comp46152_c0, and comp47135_c0, comp14825_c0, comp45314_c0) validated by q-PCR showed an expression profile inversely related to that of their miRNAs (novel-mir-6, novel-mir-12, novel-mir-52, and novel-mir-43), indicating that these genes were regulated by miRNA under Cd stress. Such regulation can be regarded as a valid evidence for the role of these miRNAs in mediating the target gene expression in response to Cd stress in ramie. The prediction of function revealed that comp46152_c0, comp45314_c0, and comp14825_c0, which are the targets of novel-mir-52, are involved in the binding of zinc ion (GO:0008270), iron (GO:0005506), and calcium (GO:0005509), respectively. These data suggest that novel-mir-52 might play a regulatory role in the uptake of cations in ramie.

Zinc (Zn^2+^) and calcium (Ca^2+^) are two essential elements for plant growth, although at very high concentrations they exert cytotoxic effects [36, 37]. Taspinar et al. [38] suggested that Zn^2+^ could inhibit the toxicity of Cd^2+^ in plants via inhibition or competition of metallothionein or via its protective role in oxidative stress. Because Ca^2+^ and Cd^2+^ have similar binding sites, the mechanism of protective action of Ca^2+^ can be related to competitive inhibition or substitution, and through the activation of Ca-ATPase. In the present study, some genes encoding Zn^2+^ binding (GO:0008270), ATP-dependent zinc metalloprotease FTSH 2, and Ca^2+^ binding (GO:0005509) were targeted by the Cd stress-responsive miRNAs, which was consistent with the results of previous studies and explained, to some extent, the high tolerance of ramie to Cd stress.

Visible symptoms of Cd injury in plants include chlorosis, stunted growth, browning of the roots, and even plant death [39, 40]. We observed that Cd treatment led to leaf chlorosis, browning of the roots, and to a slightly lower growth of ramie; all these results were consistent with those of previous studies. Because Cd can decrease the uptake of nutrients, such as manganese (Mn), iron (Fe), and magnesium (Mg), higher amounts of cellular Cd interfere with the insertion of Mg^2+^ into protoporphyrinogen or might cause the destruction of chlorophyll as a consequence of substitution of Mg^2+^ in chlorophyll *a* and *b* [41]. In the present study, the Mg transporter, MRS2–2, encoded by one of the target genes of novel-mir-51 (GO:0015693) and functioning in Mg^2+^ transmembrane transporter activity, was up-regulated by Cd stress. Another target gene associated with Mg^2+^ binding (GO:0000287) was down-regulated by Cd stress. In addition, some target genes were involved in chlorophyll biosynthesis (GO:0015995), chloroplast organization (GO:0009658), and chloroplast fission (GO:0010020). Overall, these results help to explain why the chlorophyll content of ramie leaves was lower in plants under Cd stress than in control plants.

The E3 ubiquitin-protein ligase, encoded by one of the target genes of novel-mir-51 (GO:0004842), plays a crucial role in regulating plant responses to abiotic stresses by modulating the abundance of key downstream stress-responsive transcription factors [42]. In the present study, novel-mir-51 was up-regulated by Cd stress, and therefore, E3 ubiquitin ligase might be down-regulated. This result was not consistent with that of Liu et al. [17], who showed that E3 ubiquitin-protein ligase, encoded by one of the target genes of the heat stress–responsive sja-miR2926 in *Saccharina japonica*, might be up-regulated because of the reduced expression of its miRNA. A possible reason for this discrepancy could be the fact that E3 ubiquitin-protein ligase exhibits different patterns of regulation under different abiotic stresses, as well as in different species. Taken together, these results imply that the ubiquitin proteasome system might play an important role in Cd stress response in ramie.

The abiotic stress response in plants is complex, involving perception mechanisms, signal transduction pathways, activation of stress regulatory genes, and synthesis of diverse regulatory proteins [43]. In the present study, we identified some pathways that might be involved in abiotic stress response, such as those of arginine and proline metabolism (ko00330), protein processing in endoplasmic reticulum (ko04141), and plant–pathogen interaction (ko04626). These pathways are involved in the synthesis, processing, and degradation of protein, amino acid metabolism, carbon and carbohydrate metabolism, pyrimidine, purine, and RNA metabolism, as well as in the biosynthesis of secondary metabolites. These results indicate that Cd stress triggers a complex response in ramie, but an in-depth investigation is needed to uncover the underlying mechanisms.

## Conclusions

In the present study, sRNA libraries of Cd stressed and non-stressed leaves and roots of ramie were constructed. As a result, 73 novel miRNAs were identified by high throughput sequencing, and 426 potential miRNA targets were predicted in silico. The reliability of sRNA sequencing and the relationship between the expression levels of miRNAs and their target genes were confirmed by q-PCR. The predicted function of target genes are involved in multiple pathways, including the absorption of metal ions, protein ubiquitination, and chlorophyll biosynthesis. The results indicated a set of candidate miRNAs and target genes with potential functions in regulating Cd tolerance, which would be useful for research on Cd tolerance and would provide deeper insights into the mechanisms of Cd adaptation and tolerance in ramie.

## Methods

### Plant materials and cd stress treatment

The ramie variety Zhongzhu NO.1, which demonstrates strong Cd tolerance, was used in the present study. Cottage seedlings of Zhongzhu NO.1 were planted at the Yuanjiang experimental station of Chinese Academy of Agricultural Sciences, Institute of Bast Fiber Crops (central south China). After 60 days, healthy plants were collected and the soil attached to their roots was washed away with tap water. These plants were transplanted to a hydroponic device and cultured in 1/4 Hoagland solution under the following conditions: 300 μmol m^− 2^ s^− 1^ light; 12 h light/12 h dark period; 26 °C; and 70% relative humidity. After 20 days of pre-cultivation, Cd treatment (treated with 10 mg L^− 1^ CdCl_2_) was applied. After 20 days of treatment, the leaves and roots of the control (not exposed to Cd) and CdCl_2_ treated plants were separately harvested for Illumina sequencing and quantitative PCR (q-PCR). Three independent biological replicates were set for the control and Cd treatments. Each replicate was collected from 10 randomly selected ramie plants.

### Phenotype analysis and determination of cd content

After 20 days of Cd treatment, Cd content, plant height, root length, leaf width, leaf length, biomass, and chlorophyll content were measured. For determination of Cd content, plants were dried and used to estimate Cd content using a SOLAAR M6 atomic absorption spectrometer (Thermo Fisher Scientific, MA, USA), according to the methods described by She et al. [10].

The top fourth leaf was used for chlorophyll content determination. Fresh leaves (0.01–0.02 g) were collected and immediately soaked in 95% ethanol, under dark conditions. The chlorophyll content was determined according to the methods described by Sales et al. [21].

### RNA extraction and purification

For deep sequencing, total RNA was isolated from the control and Cd treated plants within four types of samples: Cd-free leaves (as control of leaf, CL), Cd-treated leaves (treated leaf, TL), Cd-free roots (as control of root, CR), and Cd-treated roots (TR), EASYspin Plant microRNA Kit (Aidlab, RN40, Beijing, China) was used for RNA extraction. RNA quality and quantity were determined using a Nanodrop 2000 spectrophotometer (Thermo Fisher Scientific, Wilmington, DE, USA) and an Agilent 2100 Bioanalyzer (Agilent Technologies, Waldbronn, Germany).

For q-PCR, total RNA was extracted from the above-mentioned types of samples using Trizol (Roche, Mannheim, Germany), treated with RNase-free Dnase I (Takara, Dalian, China), and further purified using the RNeasy kit (Qiagen, Crawley, UK).

### Construction of small RNA library and deep sequencing

Small RNA libraries were constructed (for CL, TL, CR, and TR samples) based on high purity (OD260/280 between 1.8 and 2.2) and high integrity (RNA integrity number > 8.0) RNA using the TruSeq Small RNA Sample Preparation Kit (Illumina, RS-200–0012). The sRNAs were 5′ and 3’ RNA adapter-ligated through the T4 RNA ligase (Fermentas, Thermo Fisher Scientific, Pittsburgh, PA USA) at each step, and the resulting adapter-ligated sRNAs were subsequently transcribed into complementary DNA (cDNA) and amplified by PCR. The amplified cDNA constructs were then purified by urea polyacrylamide gel electrophoretic separation, and the four libraries recovered from this procedure were deep sequenced in a HiSeq 2500 platform according to the manufacturer’s protocol (Illumina).

### Identification and expression of miRNAs

Raw reads were processed to remove adaptors, and those with more than 20% of bases with quality score inferior to Q30 and more than 10% undetermined bases were filtered out. Reads with length above 30 nucleotides (nt) or below 18 nt were subsequently filtered out. The resulting reads were then mapped to various RNA databases, including Silva, GtRNAdb, Rfam, and Repbase by Bowtie (version 1.2.0, http://bowtie-bio.sourceforge.net/index.shtml) to exclude rRNAs, tRNAs, snRNAs, snoRNAs, and other non-coding RNAs. The remaining reads were mapped onto a previous transcriptome assembly [22] by miRDeep2 (http://mdc.helmholtz.de). Mapped reads were searched against the miRBase 21.0 (http://www.mirbase.org/) database to identify putative known miRNAs. The remaining unannotated sRNA sequences were analysed by miRDeep2 software to predict potential novel miRNAs by exploring hairpin structure, Dicer cleavage sites, and the minimum free energy. The criteria used for novel miRNA characterization were based on the work of Meyers et al. [23].

The expression level of miRNAs in terms of transcripts per million (TPM) was defined as the number of reads mapped to miRNA × 10^6^ divided by the number of reads mapped to the reference genome. Differential expression analysis between any two samples was performed using the R package DEGseq [24]. The criteria **|**log2 (fold change)**| ≥** 1 and Benjamini–Hochberg false discovery rate corrected *P*-value < 0.01 were set as thresholds for statistically significant differential expression.

### Prediction and functional annotation of novel miRNA target genes

Potential miRNA targets were identified in ramie transcripts using TargetFinder (http://carringtonlab.org/resources/targetfinder). Based on gene identification, we retrieved the sequences of miRNA targets from the National Center for Biotechnology Information (NCBI). Functional annotations of target genes were performed by sequence comparison against NCBI non-redundant (NR, ftp://ftp.ncbi.nih.gov/blast/db/), Swiss-Prot (http://www.uniprot.org/), Gene Ontology (GO, http://www.geneontology.org/), Clusters of Orthologous Groups (COG, http://www.ncbi.nlm.nih.gov/COG/), Kyoto Encyclopedia of Genes and Genomes (KEGG, http://www.genome.jp/kegg/), EuKaryotic Orthologous Groups (KOG, ftp://ftp.ncbi.nih.gov/pub/COG/KOG/), and Pfam (http://pfam.xfam.org/) databases.

### Quantitative PCR verification for cd stress–responsive miRNAs

Total RNA was reverse transcribed using the Specific Stem-loop RT Primer Kit (Acebiox, PAMIR, Shanghai, China) and q-PCR of the miRNAs was performed using the PCR Master Mix CTB103 (Acebiox, Shanghai, China). The primers of miRNAs were purchased from Acebiox Co., Ltd. (Shanghai, China). The *18S* rRNA (primer-F: ATGATAACTCGACGGATCGC, primer-R: CTTGGATGTGGTAGCCGTTT) was used as an internal control to normalize q-PCR data [25]. The internal control *18S* rRNA was reverse transcribed in parallel by oligo (dT) as RT primer (Thermo Fisher Scientific).

For target genes, total RNA was reverse transcribed using the M-MLV reverse transcriptase (Takara), and the q-PCR was performed using the PCR Master Mix CTB103 (Acebiox, Shanghai, China). The primers of target genes were purchased from Acebiox Co., Ltd. (Shanghai, China). The *18S* rRNA gene (primer-F: TGACGGAGAATTAGGGTTCGA, primer-R: CCGTGTCAGGATTGGGTAATTT) was used as the internal control to normalize q-PCR data.

All q-PCRs were performed in the Lightcycler 480 (Roche) according to the manufacturer’s instructions. Expression of miRNA/target genes was defined from the threshold cycle, and relative expression levels were calculated using the 2^-ΔΔCt^ method, after normalization based on the reference gene.

### Statistical analysis

Calculations were performed in Excel 2010 (Microsoft, Redmond, Washington, USA), and the results are presented as means ± standard deviation (SD). Statistical analysis was conducted using one-way analysis of variance (ANOVA) with SPSS Statistics 19.0 (SPSS Inc., Chicago, IL, USA). Comparisons of means were performed using the least significant difference (LSD) test at *P* = 0.05. A difference between means was considered statistically significant when *P* < 0.05.

## Additional files

Additional file 1: **Table S1.** Statistic data of high-throughput sequencing. **Table S2.** Pathway and putative function of genes targeted by cadmium stress-responsive miRNAs in leaf of ramie. **Table S3.** Pathway and putative function of genes targeted by cadmium stress-responsive miRNAs in root of ramie. (DOCX 20 kb)

Additional file 2: **Sheet 1.** Sequence and ID of the 73 novel miRNAs identified in the present study. CL, Cd-free leaf (control); TL, Cd-treated leaf; CR, Cd-free root (control); TR, Cd-treated root. **Sheet 2.** Families matched by seven of the 73 novel miRNAs. **Sheet 3.** Differentially expressed novel miRNAs. The green color denotes differentially expressed miRNAs in the leaf and root samples. The yellow and red color denote unique miRNAs expressed in the leaf and root samples, respectively. **Sheet 4.** Predicted genes targeted by the novel miRNAs from the leaf library. **Sheet 5.** Predicted genes targeted by the novel miRNAs from the root library. (XLSX 5524 kb)

## Abbreviations

Cd: Cadmium
cDNA: Complementary DNA
COG: Clusters of Orthologous Groups
DCL1: DICER-LIKE1
GO: Gene Ontology
KEGG: Kyoto Encyclopedia of Genes and Genomes
KOG: EuKaryotic Orthologous Groups
miRNAs: microRNAs
NCBI: National Center for Biotechnology Information
NR: Non-redundant
nt: Nucleotides
q-PCR: Quantitative PCR
rRNA: Ribosomal RNA
siRNAs: Small interfering RNAs
snoRNA: Small nucleolar RNA
snRNA: Small nuclear RNA
sRNAs: Small RNAs
TPM: Terms of transcripts per million
tRNA: Transfer RNA

## References

[1] Ding D, Zhang LF, Wang H, Liu ZJ, Zhang ZX, Zheng YL (2009). Differential expression of miRNAs in response to salt stress in maize roots. Ann Bot.

[2] Shriram V, Kumar V, Devarumath RM, Khare TS, Wani SH (2016). MicroRNAs as potential targets for abiotic stress tolerance in plants. Front Plant Sci.

[3] Buchet JP, Lauwerys R, Roels H, Bernard A, Bruaux P, Claeys F (1990). Renal effects of cadmium body burden of the general population. Lancet.

[4] Metwally A, Safronova VI, Belimov AA, Dietz KJ (2005). Genotypic variation of the response to cadmium toxicity in *Pisum sativum* L.. J Exp Bot.

[5] Farinati S, DalCorso G, Varotto S, Furini A (2010). The *Brassica juncea* BjCdR15, an ortholog of *Arabidopsis* TGA3, is a regulator of cadmium uptake, transport and accumulation in shoots and confers cadmium tolerance in transgenic plants. New Phytol.

[6] Zhu QH, Huang DY, Liu SL, Zhou B, Luo ZC, Zhu HH (2012). Flooding-enhanced immobilization effect of sepiolite on cadmium in paddy soil. J Soils Sediments.

[7] Zhu QH, Huang DY, Liu SL, Rao ZX, Cao XL, Ren XF (2013). Accumulation and subcellular distribution of cadmium in ramie (*Boehmeria nivea* L. gaud) planted on elevated soil cadmium contents. Plant Soil Environ.

[8] Yang B, Zhou M, Shu WS, Lan CY, Ye ZH, Qiu RL (2010). Constitutional tolerance to heavy metals of a fiber crop, ramie (*Boehmeria nivea*), and its potential usage. Environ Pollut.

[9] Zhou JH, Yang QW, Lan CY, Ye ZH (2010). Heavy metal uptake and extraction potential of two *Boehmeria nivea* (L.) gaud. (ramie) varieties associated with chemical reagents. Water Air Soil Pollut.

[10] She W, Jie YC, Xing HC, Luo ZQ, Kang WL, Huang M, Zhu SJ (2011). Absorption and accumulation of cadmium by ramie (*Boehmeria nivea*) cultivars: a field study. Acta Agr Scand B-S P.

[11] Lei M, Yue QL, Chen TB, Huang ZC, Liao XY, Liu YR, Zheng GD, Chang QR (2005). Heavy metal concentrations in soils and plant around Shizuyuan mining area of Hunan Province. Acta Ecol Sin.

[12] Phillips JR, Dalmay T, Bartels D (2007). The role of small RNAs in abiotic stress. FEBS Lett.

[13] Bartel DP (2004). MicroRNAs: genomics, biogenesis, mechanism and function. Cell.

[14] Zhou ZS, Zeng HQ, Liu ZP, Yang ZM (2012). Genome-wide identification of *Medicago truncatula* microRNAs and their targets reveals their differential regulation by heavy metal. Plant Cell Environ.

[15] Ferdous J, Sanchez-Ferrero JC, Langridge P, Milne L, Chowdhury J, Brien C, Tricker P (2017). Differential expression of microRNAs and potential targets under drought stress in barley. Plant Cell Environ.

[16] Lu SF, Sun YH, Chiang VL (2008). Stress-responsive microRNAs in *Populus*. Plant J.

[17] Liu FL, Wang WJ, Sun XT, Liang ZR, Wang FJ (2015). Conserved and novel heat stress-responsive microRNAs were identified by deep sequencing in *Saccharina japonica* (Laminariales, Phaeophyta). Plant Cell Environ.

[18] Huang SQ, Peng J, Qiu CX, Yang ZM (2009). Heavy metal-regulated new microRNAs from rice. J Inorg Biochem.

[19] Huang SQ, Xiang AL, Che LL, Chen S, Li H, Song JB, Yang ZM (2010). A set of miRNAs from *Brassica napus* in response to sulfate-deficiency and cadmium stress. Plant Biotechnol J.

[20] Kwak PB, Wang QQ, Chen XS, Qiu CX, Yang ZM (2009). Enrichment of a set of microRNAs during the cotton fiber development. BMC Genomics.

[21] Sales RMP, Fries DD, Pires AJV, Bonomo P, Santos IS, Campos CN, Brito PHR, Brito MS (2013). Chlorophyll and carbohydrates in *Arachis pintoi* plants under influence of water regimes and nitrogen fertilization. Rev Bras Zootecn.

[22] Liu TM, Zhu SY, Tang QM, Yu YT, Tang SW (2013). Identification of drought stress-responsive transcription factors in ramie (*Boehmeria nivea* L. *Gaud*). BMC Plant Biol.

[23] Meyers BC, Axtell MJ, Bartel B, Bartel DP, Baulcombe D, Bowman JL (2008). Criteria for annotation of plant microRNAs. Plant Cell.

[24] Wang LK, Feng ZX, Wang X, Wang XW, Zhang XG (2010). DEGseq: an R package for identifying differentially expressed genes from RNA-seq data. Bioinformatics.

[25] Wang J, Huang JS, Hao XY, Feng YP, Cai YJ, Sun LQ (2014). MiRNAs expression profile in bast of ramie elongation phase and cell wall thickening and end wall dissolving phase. Mol Biol Rep.

[26] Qiu ZB, Hai BZ, Guo JL, Li YF, Zhang L (2016). Characterization of wheat miRNAs and their target genes responsive to cadmium stress. Plant Physiol Biochem.

[27] Kang XP, Gao JP, Zhao JJ, Yin HX, Wang WY, Zhang P, Wang RL, Xu J (2016). Identification of cadmium-responsive microRNAs in *Solanum torvum* by high-throughput sequencing. Russ J Plant Physiol.

[28] Nozawa M, Miura S, Nei M (2012). Origins and evolution of microRNA genes in *Drosophila* species. Genome Biol Evol.

[29] Cock JM, Sterck L, Rouzé P, Scornet D, Allen AE, Amoutzias G (2010). The *Ectocarpus* genome and the independent evolution of multicellularity in brown algae. Nature.

[30] Farooq M, Mansoor S, Guo H, Amin I, Chee PW, Azim MK, Paterson AH (2017). Identification and characterization of miRNA transcriptome in Asiatic cotton (*Gossypium arboreum*) using high throughput sequencing. Front Plant Sci.

[31] Leung AKL, Sharp APA (2010). MicroRNA functions in stress responses. Mol Cell.

[32] Huang CY, Shirley N, Genc Y, Shi BJ, Langridge P (2011). Phosphate utilization efficiency correlates with expression of low affinity phosphate transporters and noncoding RNA, *IPS1*, in barley. Plant Physiol.

[33] Gielen H, Remans T, Vangronsveld J, Cuypers A (2012). MicroRNAs in metal stress: specific roles or secondary responses?. Int J Mol Sci.

[34] Pilon M (2016). The copper microRNAs. New Phytol.

[35] Baumberger N, Baulcombe DC (2005). *Arabidopsis* ARGONAUTE1 is an RNA slicer that selectively recruits microRNAs and short interfering RNAs. Proc Natl Acad Sci U S A.

[36] Sinha KK, Kumari P (1990). Some physiological abnormalities induced by aflatoxin B_1_ in mung seeds (*Vigna radiata* variety Pusa Baishakhi). Mycopathologia.

[37] Jihen E, Imed M, Fatima H, Abdelhamid K (2008). Protective effects of selenium (se) and zinc (Zn) on cadmium (cd) toxicity in the liver and kidney of the rat: histology and cd accumulation. Food Chem Toxicol.

[38] Taspinar MS, Agar G, Alpsoy L, Yildirim N, Bozari S, Sevsay S (2011). The protective role of zinc and calcium in *Vicia faba* seedlings subjected to cadmium stress. Toxicol Ind Health.

[39] Schützendübel A, Schwanz P, Teichmann T, Gross K, Langenfeld HR, Godbold DL, Polle A (2001). Cadmium induced changes in antioxidative systems, hydrogen peroxide content differentiation in scots pine roots. Plant Physiol.

[40] Rizwan M, Ali S, Adrees M, Rizvi H, Zia-ur-Rehman M, Hannan F, Qayyum MF, Hafeez F, Ok YS (2016). Cadmium stress in rice: toxic effects, tolerance mechanisms management: a critical review. Environ Sci Pollut Res Int.

[41] Gillet S, Decottignies P, Chardonnet S, Maréchal PL (2006). Cadmium response and redoxin targets in *Chlamydomonas reinhardtii*: a proteomic approach. Photosynth Res.

[42] Lyzenga WJ, Stone SL (2012). Abiotic stress tolerance mediated by protein ubiquitination. J Exp Bot.

[43] Wahid A, Gelani S, Ashraf M, Foolad MR (2007). Heat tolerance in plants: an overview. Environ Exp Bot.

