# Supplementary material for: The miRNAome of ramie (Boehmeria nivea L.): identification, expression, and potential roles of novel microRNAs in regulation of cadmium stress response
Source: BMC Plant Biol. 2018 Dec 22;18:369. doi: 10.1186/s12870-018-1561-5 (PMC6303851; doi:10.1186/s12870-018-1561-5)
Supplement: Supplementary file 1 — Table S1. Statistic data of high-throughput sequencing. Table S2. Pathway and putative function of genes targeted by cadmium stress-responsive miRNAs in leaf of ramie. Table S3. Pathway and putative function of genes targeted by cadmium stress-responsive miRNAs in root of ramie. (DOCX 20 kb) [file 12870_2018_1561_MOESM1_ESM.docx]

**The miRNAome of ramie (*Boehmeria nivea* L.): identification, expression, and potential roles of novel microRNAs in regulation of cadmium stress response**

**Kunmei Chen^+^, Yongting Yu^+^, Kai Sun, Heping Xiong, Chunming Yu*, Ping Chen, Jikang Chen, Gang Gao, and Aiguo Zhu***

* Corresponding authors.

**^+^** these authors contributed equally to the article.

**Supplementary Table S1.** statistic data of high-throughput sequencing

| Samples | Raw reads | Low quality reads | Containing 'N' reads | <18 nt reads | >30 nt reads | Clean reads | Q30(%) | mapped reads | percentage  (%) |  |
| --- | --- | --- | --- | --- | --- | --- | --- | --- | --- | --- |
| CL | 32557284 | 0 | 1917 | 2012340 | 18200867 | 12342160 | 95.07 | 1941679 | 26.21 |  |
| TL | 34638385 | 0 | 2941 | 4209340 | 16401918 | 14024186 | 93.25 | 1787093 | 23.03 |  |
| CR | 29537747 | 0 | 21995 | 6740545 | 3049945 | 19725262 | 91.62 | 657206 | 9.48 |  |
| TR | 21208181 | 0 | 15560 | 4449003 | 1756565 | 14987053 | 92.15 | 550022 | 11.62 |  |

**Supplementary Table S2.** Pathway and putative function of genes targeted by cadmium stress-responsive miRNAs in leaf of ramie

| KEGG Pathway | ko_ID | KO | Putative function of target genes |
| --- | --- | --- | --- |
| Arachidonic acid metabolism | ko00590 | K00681 | Gamma-glutamyltranspeptidase 1 |
| Glyoxylate and dicarboxylate metabolism | ko00630 | K01601 | Ribulose bisphosphate carboxylase large chain |
| Arginine and proline metabolism | ko00330 | K12657 | Gamma-glutamyl phosphate reductase |
| Taurine and hypotaurine metabolism | ko00430 | K00681 | Gamma-glutamyltranspeptidase 1 |
| mRNA surveillance pathway | ko03015 | K14401 | Cleavage and polyadenylation specificity factor subunit 1 |
| Base excision repair | ko03410 | K01246 | Putative Glutamine amidotransferase |
| Cyanoamino acid metabolism | ko00460 | K00681 | Gamma-glutamyltranspeptidase 1 |
| RNA transport | ko03013 | K03259 | Eukaryotic translation initiation factor isoform |
| Carbon fixation in photosynthetic organisms | ko00710 | K01601 | Ribulose bisphosphate carboxylase large chain |
| Glutathione metabolism | ko00480 | K00681 | Gamma-glutamyltranspeptidase 1 |
| Glycerolipid metabolism | ko00561 | K00679 | Phospholipid:diacylglycerol acyltransferase 1 |

**Supplementary Table S3.** Pathway and putative function of genes targeted by cadmium stress-responsive miRNAs in root of ramie

| KEGG Pathway | ko_ID | KO | Putative function of target genes |
| --- | --- | --- | --- |
| Base excision repair | ko03410 | K10773 | Replication, recombination and repair |
| Phagosome | ko04145 | K07342 | Intracellular trafficking, secretion, and vesicular transport |
| Glycerolipid metabolism | ko00561 | K00679 | Lipid transport and metabolism |
| Valine, leucine and isoleucine degradation | ko00280 | K00167 | Energy production and conversion |
| Taurine and hypotaurine metabolism | ko00430 | K00681 | Amino acid transport and metabolism |
| RNA transport | ko03013 | K03259, K03257, K03243, K12881 | Translation, ribosomal structure and biogenesis, RNA processing and modification |
| Glutathione metabolism | ko00480 | K00681 | Amino acid transport and metabolism |
| Protein processing in endoplasmic reticulum | ko04141 | K07342 | Intracellular trafficking, secretion, and vesicular transport |
| Spliceosome | ko03040 | K12881 | RNA processing and modification |
| mRNA surveillance pathway | ko03015 | K12881 | RNA processing and modification |
| Citrate cycle (TCA cycle) | ko00020 | K01648 | Energy production and conversion |
| Glycosylphosphatidylinositol(GPI)-anchor biosynthesis | ko00563 | K05293 | General function prediction only |
| Cyanoamino acid metabolism | ko00460 | K00681 | Amino acid transport and metabolism |
| Purine metabolism | ko00230 | K00860 | Inorganic ion transport and metabolism |
| Glycosaminoglycan degradation | ko00531 | K01205 | Intracellular trafficking, secretion, and vesicular transport |
| Arachidonic acid metabolism | ko00590 | K00681 | Amino acid transport and metabolism |
| Terpenoid backbone biosynthesis | ko00900 | K00099 | Lipid transport and metabolism |
| Arginine and proline metabolism | ko00330 | K12657 | Amino acid transport and metabolism |
| Protein export | ko03060 | K07342 | Intracellular trafficking, secretion, and vesicular transport |
| Starch and sucrose metabolism | ko00500 | K01087 | Carbohydrate transport and metabolism |
| Carotenoid biosynthesis | ko00906 | K06444 | carotenoid biosynthetic process |
| Glycerophospholipid metabolism | ko00564 | K01613 | Lipid transport and metabolism |
| Sulfur metabolism | ko00920 | K00860 | Inorganic ion transport and metabolism |
| Plant-pathogen interaction | ko04626 | K13448, K13457 | Signal transduction mechanisms |
| Endocytosis | ko04144 | K12489 | Signal transduction mechanisms |
